# Reports from the Laboratory of the Royal College of Physicians, Edinburgh

**Published:** 1889-03

**Authors:** 


*Reports from the Laboratory of the Royal College of Physicians, Edinburgh.* Edited by J. BATTY TUKE, M.D., and G. SIMS WOODHEAD, M.D. Vol. I., 1889. Edinburgh and London: YOUNG J. PENTLAND.

We heartily welcome this first volume of Reports from the laboratory recently established by the Royal College of Physicians of Edinburgh, not only on the ground of the excellence of the material it contains, but also because it signalises a new departure in the history of a great corporation, which is destined not only to benefit the profession, but to serve as an example to other corporations. We sincerely congratulate the Edinburgh College of Physicians on the success of their effort. We scarcely know which to praise most: the skill and acumen with which the laboratory has been fitted up; the small expense it has involved; or the great principle it has put into practice. This laboratory will certainly be a notable increment to the educational influence of Edinburgh as a medical centre.

Alas! that by comparison, congratulation and encouragement should suggest depression and condemnation! Here are we in England observing a small and comparatively poor corporation in Scotland founding a laboratory; encouraging and partly endowing research; educating individuals, and advancing science; while we see our greatest corporation, the curators of the finest museums in the world, recently endowed with enormous wealth, crushing popular demands, spending its revenues in legal enforcement of foolish claims, and contemptuously setting aside the clamours of thinking and working men for the establishment of laboratories and the encouragement of research.

